# Cyclopedia of Practical Medicine

**Published:** 1844-06

**Authors:** 


### BIBLIOGRAPHICAL NOTICES.

*Cyclopedia of Practical Medicine.* Edited by John Forbes, M. D., F. R. S., Alexander Tweedie, M. D., F. R. S., and John Conolly, M. D. Revised with additions. By Robley Dunglison, M. D. Part I. Lea & Blanchard, Phila.

This is one of the most valuable works of reference that these well known publishers have issued for many years. Already a standard in England, it must become the same in this country.—"The work comprises nearly 300 *original essays* contributed by no less than 67 of the most eminent practical Physicians of Great Britain and Ireland, and among them many of the Professors and Teachers in London, Edinburgh, Dublin, and Glasgow, whose reputation conveys a high and just authority to their doctrines. Each subject has been treated by a writer of acknowledged eminence, whose particular studies have eminently fitted him for the task; and all the articles are authenticated with the names of the author." Dr. Dunglison's additions, will, we doubt not, add much to the value of the work.

It will be comprised in twenty-four parts, (at fifty cents each,) one of which is to be issued every two weeks; so that the work will be completed during the present year.—Ed.

